# Astaxanthin Inhibits Mitochondrial Permeability Transition Pore Opening in Rat Heart Mitochondria

**DOI:** 10.3390/antiox8120576

**Published:** 2019-11-21

**Authors:** Yulia Baburina, Roman Krestinin, Irina Odinokova, Linda Sotnikova, Alexey Kruglov, Olga Krestinina

**Affiliations:** 1 Laboratory of Pharmacological Regulation of Cell Resistance, Institute of Theoretical and Experimental Biophysics, Russian Academy of Sciences, Moscow Region, 142290 Pushchino, Russia; byul@rambler.ru (Y.B.); rkrestinin@bk.ru (R.K.); odinokova@rambler.ru (I.O.); linda_sotnikova@mail.ru (L.S.); krugalex@rambler.ru (A.K.); 2 Department of Biophysics and Medicobiological Sciences, Pushchino State Natural Science Institute, Moscow Region, 142290 Pushchino, Russia

**Keywords:** mitochondria, permeability transition, non-specific pore, regulatory proteins of mPTP, cell signaling, astaxantin (AST) administration

## Abstract

The mitochondrion is the main organelle of oxidative stress in cells. Increased permeability of the inner mitochondrial membrane is a key phenomenon in cell death. Changes in membrane permeability result from the opening of the mitochondrial permeability transition pore (mPTP), a large-conductance channel that forms after the overload of mitochondria with Ca^2+^ or in response to oxidative stress. The ketocarotenoid astaxanthin (AST) is a potent antioxidant that is capable of maintaining the integrity of mitochondria by preventing oxidative stress. In the present work, the effect of AST on the functioning of mPTP was studied. It was found that AST was able to inhibit the opening of mPTP, slowing down the swelling of mitochondria by both direct addition to mitochondria and administration. AST treatment changed the level of mPTP regulatory proteins in isolated rat heart mitochondria. Consequently, AST can protect mitochondria from changes in the induced permeability of the inner membrane. AST inhibited serine/threonine protein kinase B (Akt)/cAMP-responsive element-binding protein (CREB) signaling pathways in mitochondria, which led to the prevention of mPTP opening. Since AST improves the resistance of rat heart mitochondria to Ca^2+^-dependent stress, it can be assumed that after further studies, this antioxidant will be considered an effective tool for improving the functioning of the heart muscle in general under normal and medical conditions.

## 1. Introduction

Mitochondrial dysfunction is a cause of various diseases, including neurodegenerative and cardiovascular diseases, diabetes, different forms of damage to the liver and the skeletal and muscle systems, sepsis, and psychiatric disorders [1]. Oxidative stress and impairment of Ca^2+^ homeostasis are considered to be important factors in mitochondrial dysfunction, which results in the development of programmed cell death [2]. Disturbances in the functional states of mitochondria are also observed during the development of myocardial infarction, the most commonly encountered ischemic heart disease in the world, a cause of premature death in humans [3]. Mitochondria play a key role in the physiological function of the heart, and in the pathogenesis and progression of various heart diseases. Stores of mitochondrial ATP usually correlate with changes in ATP uptake by the heart, largely mediated through Ca^2+^ transport pathways [4]. Mitochondrial Ca^2+^ plays an important role in the generation of reactive oxygen species (ROS) and the opening of mitochondrial permeability transition pore (mPTP), factors involved in the development of both ischemia/reperfusion and heart failure [4,5,6]. A nonspecific increase in the permeability of the inner membrane, which leads to the formation of mPTP in mitochondria, may be the cause of mitochondrial dysfunction and cell death. The composition of pore is not yet clearly established; therefore, by convention, the supposed structural components of mPTP are considered to be pore regulators. Among these are the voltage-dependent anion channel (VDAC) and the translocation protein (TSPO), which are localized in the outer mitochondrial membrane; adenine nucleotide translocase (ANT) of the inner membrane; cyclophilin D (CyP-D) and the phosphate carrier in the matrix; creatine kinase, localized in the intermembrane space; and hexokinase, localized in the cytosol [6]. We previously detected a protein in nonsynaptic, purified mitochondria from rat brains, which was identified as 2′,3′-cyclic nucleotide-3′-phosphodiesterase (CNPase). CNPase is a myelin protein, which is also found in non-myelin-forming tissues [7,8,9]. We have shown that CNPase participates in the regulation of mPTP opening. Moreover, CNPase is colocalized with CyP-D, VDAC, ANT, and α-tubulin [8]. Subunit *c*, the mitochondrial (*N*,*N*-dicyclohexylcarbodiimide (DCCD)-binding proteolipid [10], also known as subunit 9 F_0_c, forms in cooperation with subunit *a*, the proton channel of F_o_F_1_-ATPase [11]. In mammals, subunit *c* of F_o_F_1_-ATP synthase has three isoforms (P1, P2, and P3), encoded by *ATP5G1*, *ATPG2*, and *ATPG3* genes [12]. Recently, we showed that subunit c of F_o_F_1_-ATPase might be a structural and/or regulatory component of the mPTP complex, whose activity might be modulated by calcium-dependent phosphorylation [13].

Alterations in mitochondrial bioenergetics play important roles in the origin and progression of myocardial ischemia [14], manifesting as inhibition of respiratory complex activity, increased proton leakage from the inner mitochondrial membrane [15], increased ROS production [16], mitochondrial calcium overload [17], and finally, opening of mPTP [18]. During the development of myocardial ischemia, oxygen deprivation alters mitochondrial function and ATP synthesis, causing an important reduction in cardiac ATP production [19]. Because calcium regulation requires ATP and mPTP remains open as ATP pools dissipate, calcium levels in the ischemic heart are further elevated [18]. In addition to Ca^2+^ elevation, ROS production is also increased in the ischemic heart [20]. Thus, the ischemic heart is primed for a prolonged mPTP opening cycle that ultimately leads to cardiomyocyte death [20]. Ischemic conditioning as cardioprotection is believed to prevent mPTP opening at reperfusion by modulating factors known in mPTP opening, such as cellular energetic status, mitochondrial Ca^2+^ overloading, ROS production, and rapid pH correction either directly or indirectly via known cardioprotective signaling pathways. The identification of the molecular pore of the mPTP should provide further insight into unravelling the signaling pathway involved in inhibition of mPTP and further enhance the translatability of mPTP inhibition to the clinical setting [21]. mPTP has become an obvious target for cardioprotection. Drugs that inhibit mPTP directly can be of great importance in protecting the heart during heart surgery or in the treatment of coronary thrombosis.

To reduce both oxidative damage and the development of various heart diseases, considerable attention has been recently focused on studies aimed at enhancing the protective response of the organism to oxidative stress using different antioxidants. Of some interest among antioxidants is astaxanthin (AST). AST is a red pigment that belongs to xanthophylls, a subclass of carotenoids. AST possesses strong antioxidant capacity and can absorb singlet oxygens and free radicals [22]. It is found in algae, yeasts, and sea animals, such as salmon, trout, common shrimp, and lobster. Structurally, an AST molecule represents a long polyene skeleton with polar ionone rings at the ends. As a result of long-term coupling, AST responds to reduction by free radicals, and the presence of polar hydroxyl and carbonyl-containing ionone rings endows it with higher antioxidant capacity compared with other carotenoids [23]. Owing to the polar-nonpolar-polar linear structural matrix, AST can bind to the cell membrane [24]. It is also known that AST considerably attenuates the mitochondrial dysfunction associated with ischemic myocardial injury [25]. In particular, it restores the integrity of mitochondria and inhibits mitochondrion-mediated apoptosis in a model of homocysteine-induced cardiotoxicity [26]. 

A crucial regulatory system of signal transduction that controls many aspects of cellular functions is protein phosphorylation/dephosphorylation. One important protein kinase found in mitochondria is serine/threonine protein kinase B (Akt) [27]. The results of studies have indicated that the targets of mitochondrial Akt are complex V and hexokinase-II [28,29]. In addition, Akt directly affects mitochondria, which leads either to their protection against oxidants or to mPTP opening induced by Ca^2+^ [28,29]. Recently, it was supposed that Akt is present in mitochondria and its level is dynamically regulated by cellular signaling activities. Within the mitochondria, Akt phosphorylates the β-subunit of ATP synthase, GSK3β, and other unidentified proteins [30]. 

It is known that protein kinase A (PKA) phosphorylates cAMP-responsive element-binding protein (CREB) at Ser-133; the localization of PKA and CREB in mitochondria contributes to their possible involvement in cell survival by the regulation of mitochondrial functions through mitochondrial PKA and CREB signaling pathways [31]. Furthermore, it was shown that CREB can be phosphorylated by Akt at Ser133, leading to activation of its transcription and inhibition of apoptosis [32]. CREB plays a role in regulating the expression of some mitochondrial proteins, such as respiratory chain subunits [33], cytochrome *c* [34], manganese superoxide dismutase [35], and carnitine palmitoyltransferase [36]. Activated CREB is able to inhibit mitochondrial activity in several cell lines and acts as a survival and differentiating factor. There is a hypothesis that CREB also plays a role in impaired mitochondrial activity [37]. Arnold and coauthors found that phosphorylated CREB induced by mitochondrial dysfunction can be considered a factor causing a defect of the cell cycle; in cells with disturbed oxidative phosphorylation, CREB activation induced by mitochondrial dysfunction is a new signaling pathway that impairs cell proliferation [38].

The goal of the present work was to study the effect of AST on the functional state of rat heart mitochondria (RHM) under the conditions of mPTP opening and mitochondrial swelling by both direct addition of AST to mitochondria and AST administration. In addition, we wanted to check how AST changes the level of regulatory proteins of mPTP. We studied the involvement of AST in Akt/CREB signaling pathways in mitochondria under mPTP opening.

## 2. Materials and Methods 

### 2.1. Animals and Treatment

Five 2-month-old male Wistar rats (240–250 g weight) were used in the experiments. For each separate experiment, one rat was used; thus, 5 independent replicates were done. Animals were individually housed in a temperature-controlled room (22 °C) and kept on a standard diet with free access to water and food. All experiments were performed in accordance with the Regulations for Studies with Experimental Animals (Decree of the Russian Ministry of Health of 12 August 1997, No. 755). The protocol was approved by the Commission on Biological Safety and Ethics at the Institute of Theoretical and Experimental Biophysics, Russian Academy of Sciences (March 2019, protocol N18/2019).

For AST administration, we used 2 groups of rats (5 rats in each group). The first group of rats was the control group, and the second group was treated with AST. AST was administered orally using plastic feeding tubes, 15 ga × 78 mm (Instech, Plymouth Meeting, PA, USA). The second group of rats was treated with food supplemented with 150 mg/kg of 5% astaxanthin (Natural, China) orally every day for 2 weeks. A weighed quantity of AST was dissolved in olive oil, which was chosen as the vehicle because AST is soluble in olive oil. For purity of the experiment, the control group of rats received olive oil in the same volume as the experimental group. After 2 weeks, RHM were isolated from the hearts of rats in each group.

### 2.2. Isolation of Rat Heart Mitochondria

Mitochondria were isolated from the whole hearts by the standard technique [39]. Each isolated heart was chopped, cleared of blood vessels, and destroyed with a glass homogenizer in a 10-fold volume of a medium containing 75 mM sucrose, 10 mM Tris-HCl (pH 7.4), 225 mM mannitol, 1 mM EDTA, and 0.1% BSA at 4 °C. The homogenate was centrifuged at 1000× *g* for 10 min, and the pellet was removed. Mitochondria contained in the supernatant were sedimented at 6000× *g* for 10 min at 4 °C. Then, the mitochondrial pellet was washed with the isolation medium without EDTA and BSA (6000× *g*, 10 min) and suspended in the same medium. The protein content in mitochondria was determined using the Bradford assay. Protein concentration in RHM suspension was 30–35 mg/mL. Isolated mitochondria were kept for 2 h at 4 °C.

### 2.3. Evaluation of Mitochondrial Functions

In the experiments, mitochondria were preincubated with AST (Sigma, USA) for 10 min, after which mitochondria with AST were added to the multifunctional chamber with the incubation medium and incubated until the addition of Ca^2+^ for 3–5 min. The Ca^2+^ flow and Ca^2+^ retention capacity (CRC) of RHM were determined with TPP^+^- and Ca^2+^-sensitive electrodes (Nico, Russia), and the oxygen consumption rate was measured with a Clark-type O_2_ electrode in a 1 mL measuring chamber [40]. Mitochondria (1 mg protein/mL) were incubated in a medium containing 125 mM KCl, 10 mM Tris (pH 7.4), and 2 mM K_2_HPO_4_ at 25 °C. In the experiments, glutamate (5 mM) and malate (5 mM) were used as respiratory substrates. The respiratory control index (RCI) was measured in a closed chamber after the addition of 150 μM ADP to RHM. mPTP opening in RHM was induced by a threshold [Ca^2+^] concentration (first addition of Ca^2+^ contained 50 nM per mg of protein; the next additions of Ca^2+^ were 100 nM per mg of protein). A threshold [Ca^2+^] concentration is the concentration of Ca^2+^ added to a mitochondrial suspension at which Ca^2+^ ions (when accumulating in mitochondria) induce mPTP opening. Parameters of Ca^2+^ transport, such as rates of influx (V^Ca2+^_in_, nmol min^−1^ mg^−1^ of protein), efflux (V^Ca2+^_out_, nmol min^−1^ mg^−1^ protein), and lag-phase (time between influx and efflux, ΔTlag, s) were measured. V^Ca2+^_in_ is the rate of Ca^2+^ influx during the last Ca^2+^ addition. V^Ca2+^_out_ is the rate of Ca^2+^ efflux after Ca^2+^ release. Lag-phase shows the time between influx and efflux during the last Ca^2+^ addition. The Ca^2+^-induced dissipation of membrane potential was calculated as TPP^+^ efflux rate (V^TPP+^_out_, nmol min^−1^ mg^−1^ of protein).

RHM swelling was measured as a change in light scattering in a mitochondrial suspension at 540 nm (A540) using a Tecan I-Control Infinite 200 spectrophotometer at 25 °C. The standard incubation medium for the swelling assay contained 125 mM KCl, 10 mM Tris, 2 mM KH_2_PO_4_, 5 mM glutamate, and 5 mM malate. The concentration of mitochondrial protein in a well was 0.5 mg protein/mL. Swelling was initiated by the addition of 260 nmol of Ca^2+^ per mg of protein. The swelling process was characterized by the time needed to reach the half-maximal light scattering signal (T_1/2_).

### 2.4. The Sample Preparation, Electrophoresis, and Immunoblotting of Mitochondrial Proteins

To prepare samples for the determination of regulator protein levels, aliquots of isolated intact RHM from each group were placed in an Eppendorf tube and solubilized in Laemmli buffer (Bio-Rad, USA). Then, the samples were heated to 95 °C for 3 min. To determine pAKT and pCREB levels, 50 μL aliquots were taken from the chamber and centrifuged at 15,000× *g* for 3 min. Pellets were taken and solubilized in Laemmli buffer. The samples were heated to 95 °C for 3 min. 

Samples containing 20 μg of mitochondrial protein were applied to each line and subjected to electrophoresis followed by western blot analysis. Precision Plus Pre-stained Standards from Bio-Rad Laboratories (Hercules, CA, USA) were used as markers. 

Polyclonal anti-TSPO antibody (1:1000), monoclonal anti-cyclophilin D antibody (1:1000), and monoclonal anti-ATPG1/G2/G3 antibody–subunit *c* (1:1000) were from Abcam. VDAC antibody (1:1000) was purchased from Calbiochem; polyclonal rabbit phospho-Akt (Ser473) and Akt antibodies (dilution 1:250) were from Cell Signaling; phospho-CREB (Ser133) and CREB (86B10) (dilution: 1:500) were from Cell Signaling. The COX IV antibody (1:1000 dilution; Abcam, USA) was used as the loading control. Immunoreactivity was detected using the appropriate secondary antibody conjugated to horseradish peroxidase (Jackson Immuno Research, West Grove, PA, USA). The blot was detected with ECL (Bio-Rad, Hercules, California, USA) using ChemiDoc Touch Imaging System (Bio-Rad, USA). Protein bands were quantified by densitometry (Image Lab program).

### 2.5. Statistical Analysis

For statistical analysis, relative levels of protein density were expressed as means ± SDs from at least 5 independent experiments. The statistical significance of the differences between pairs of mean values was evaluated using Student’s *t*-tests. The difference was considered significant at *p* < 0.05.

## 3. Results

### 3.1. The Effect of AST on Respiratory Activity in Rat Heart Mitochondria

**Figure 1:**
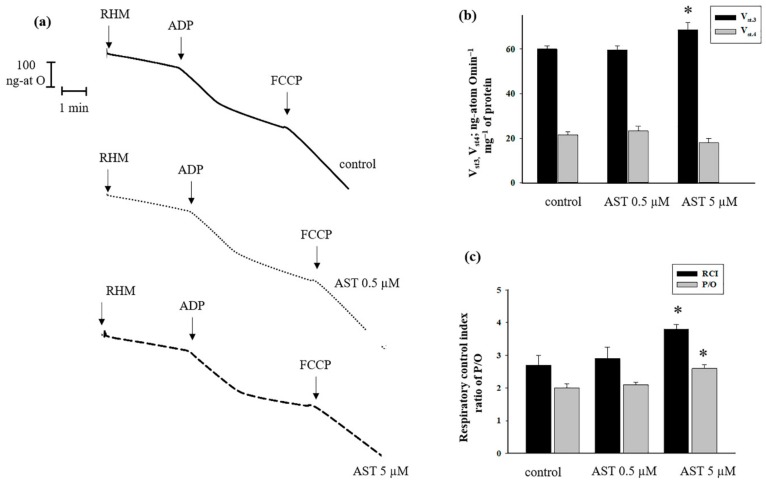
The Effect of astaxanthin (AST) on respiratory activity in rat heart mitochondria (RHM). RHM were incubated in standard medium as described in Materials and Methods. Arrows show the times at which FCCP (Carbonyl cyanide 4-(trifluoromethoxy) phenylhydrazone) and ADP (adenosine 5 ‘-diphosphate) were added. (**a**) Curves of respiratory activity; (**b**) quantitative analysis of RHM respiration rate in states 3 and 4 in the absence/presence of AST (0.5 and 5 µM); (**c**) values of respiratory control index (RCI) and phosphate/oxygen (P/O) ratio in the presence/absence of AST. Data are presented as means ± SDs of five independent experiments. * *p* < 0.05 compared with control (without AST).

First, we measured the respiratory activity of the RHM (Figure 1). The experiments were carried out as described in Materials and Methods. Figure 1a shows the curves of mitochondrial respiration in the absence or presence of two different concentrations of AST. The evaluation of oxygen consumption rates in different states is represented in Figure 1b. As seen from the figure, no changes occurred in the rate of states 3 and 4 when 0.5 µM of AST was added to RHM in comparison with control (without AST). The addition of 5 µM AST led to an increased respiration rate in state 3 by approximately 13% in comparison with control. In the presence of 5 µM AST, the respiration rate in state 4 decreased by approximately 20% relative to control. The respiratory control index (RCI) indicates the effectiveness of the mitochondria in promoting oxidative phosphorylation and coupling between oxygen consumption and ATP production. The results (Figure 1c) indicate that RHM incubated in standard medium with malate and glutamate in control conditions described in Material and Methods, exhibited an RCI of 2.7. The RCI of mitochondria in the presence of AST (0.5 µM) did not differ from control. However, treatment of mitochondria with 5 µM AST caused the RCI to increase to 3.9. Thus, 5 µM AST increased the RCI of RHM by approximately 40% compared to control. The efficiency of oxidative phosphorylation in mitochondria is defined as the ratio of ATP to absorbed oxygen. We measured this parameter in the presence of both concentrations of AST. The phosphate/oxygen (P/O) ratio of RHM in the presence of 0.5 µM AST (2) did not differ from the control value (2.1), whereas 5 µM AST increased the P/O ratio (2.73) by approximately 30%.

### 3.2. The Effects of AST on Ca^2+^ Transport and Ca^2+^-Induced PTP Opening in Rat Heart Mitochondria

**Figure 2:**
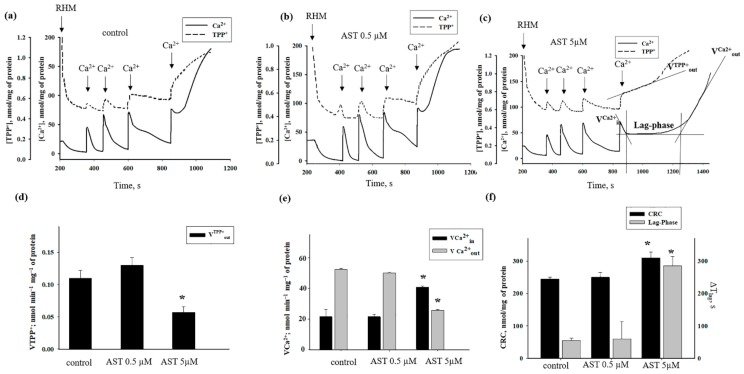
The effects of AST on Ca^2+^ transport, membrane potential, and CRC in RHM. Before being added to chamber, RHM were incubated with AST for 10 min. Before the addition of Ca^2+^, mitochondria were incubated with AST for 3 min in chamber. (**a**) Ca^2+^ transport and membrane potential changes in RHM in control; (**b**) Ca^2+^ transport and membrane potential changes in RHM in the presence of 0.5 µM AST; (**c**) Ca^2+^ transport and change of membrane potential in RHM in the presence of 0.5 µM AST; (**d**) quantitative analysis of V^TPP+^_out_; (**e**) quantitative analysis of V^Ca2+^_in_ and V^Ca2+^_out_ in RHM; (**f**) quantitative analysis of CRC and lag-phase. Data are presented as means ± SDs of five independent experiments. * *p* < 0.05 compared with control (without AST).

Because mitochondrial dysfunction induced by oxidative stress can affect morphological and functional changes in mitochondria [41], the effects of induced mPTP opening in RHM by AST (0.5 and 5 μM) on Ca^2+^ transport, membrane potential (Δψm), and calcium retention capacity (CRC) were examined. In Figure 2a–c, the curves of changes in Ca^2+^ flow in the control and in the presence of AST are shown.

The first addition of Ca^2+^ (50 nmol per mg of protein) led in all cases to a stable accumulation of Ca^2+^ in mitochondria followed by membrane potential recovery. The next additions of Ca^2+^ consisted of 100 nmol per mg of protein, and the fourth 100 nmol addition in control conditions resulted in Ca^2+^ release and mPTP opening (Figure 1a). In the presence of 0.5 µM AST (Figure 2b), changes in Ca^2+^ flow (rate of Ca^2+^ uptake and efflux) did not differ from those of control. The addition of 5 µM AST (Figure 2c) led to the activation of mPTP after the fourth addition of Ca^2+^ but after a prolonged lag-phase. In the presence of 5 µM AST, CRC was increased by 26% (Figure 2f) and the rate of TPP^+^ efflux was delayed doubly relative to the control (Figure 2d). The rates of Ca^2+^ uptake and efflux in control RHM were 21.5 nmol min^−1 mg−1^ and 52.25 nmol min^−1 mg−1^, respectively. In 5 µM AST-treated RHM, the rate of Ca^2+^ uptake was increased to 40 nmol min^-1 mg-1^ and the maximal Ca^2+^ efflux rate was diminished to 35.6 nmol min^−1 mg−1^. Thus, the Ca^2+^ influx/efflux ratio was approximately 0.4 in control mitochondria and 1.56 in 5 µM AST-treated RHM. In the presence of 5 µM AST, Ca^2+^ efflux and Δψm dissipation occurred after a prolonged lag-phase, and a significant amount of previously accumulated Ca^2+^ was retained in the mitochondrial matrix, even when Δψm was largely dissipated (Figure 2c). Thus, 5 µM AST slowed down mPTP opening.

mPTP is known to be Ca^2+^-dependent and cyclosporin A (CsA)-sensitive [42,43]. In the presence of CsA, an additional fourth pulse of Ca^2+^ was tolerated without any efflux of mitochondrial Ca^2+^ and without collapse of Δψm in all experimental conditions (data not shown). Thus, the effects observed were CsA-sensitive, indicating the involvement of mPTP in the process.

### 3.3. The Effect of AST on the Swelling of Rat Heart Mitochondria

**Figure 3:**
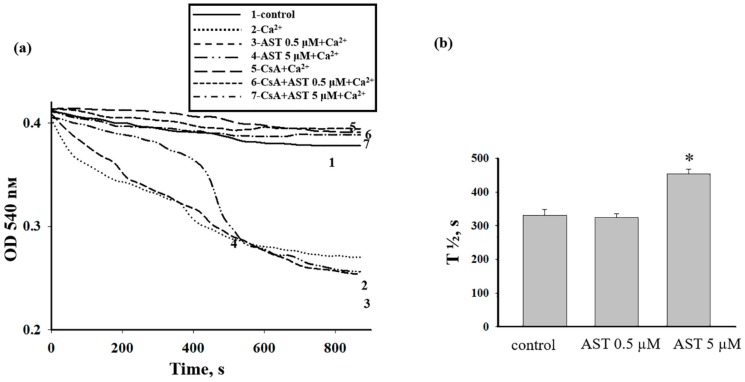
The effect of AST on Ca^2+^-induced swelling of RHM. (**a**) Curves of RHM swelling: in control conditions without Ca^2+^ (curve 1), in the presence of threshold [Ca^2+^] alone (curve 2), and with 0.5 µM AST (curve 3), 5 µM AST (curve 4), cyclosporin A (CsA) (curve 5), CsA + 0.5 µM AST (curve 6), and CsA + 5 µM AST (curve 7). (**b**) Half-maximal mitochondrial swelling (T_1/2_). Concentration of protein in a cuvette was 0.35 mg/mL. Data are presented as means ± SDs of five independent experiments. * *p* < 0.05 compared with control (without AST).

To verify the inhibitory effect of AST, we examined another parameter that characterizes mPTP opening, Ca^2+^-induced swelling of mitochondria. The addition of Ca^2+^ at a threshold concentration to mitochondrial suspension incubated in a standard medium caused a decrease in light scattering, indicating swelling, and mitochondrial membranes became permeable to low-molecular-weight substances. The amount of Ca^2+^ added to mitochondria incubated in the presence of AST was 260 nmol per mg of protein and corresponded to the control value (Figure 3). The curves of RHM swelling under different experimental conditions are shown in Figure 3a. Figure 3b quantitatively illustrates the course of the swelling process, characterized by the average half-maximal light scattering signal (T_1/2_). It is visible in the figure that after the addition of 5 µM AST to the mitochondrial suspension, the rate of mitochondrial swelling decreased by approximately 36%, whereas after the addition of 0.5 µM AST, the swelling rate did not differ from the control (threshold [Ca^2+^], curve 2). CsA, confirming the participation of permeability transition, prevented mitochondrial swelling (curves 5–7). Because 0.5 µM AST did not significantly affect the functional state of RHM and its effect did not differ from control, further studies were performed using only 5 µM AST.

### 3.4. The Effect of AST Administration on Levels of Regulated Proteins of mPTP in Intact Rat Heart Mitochondria

**Figure 4:**
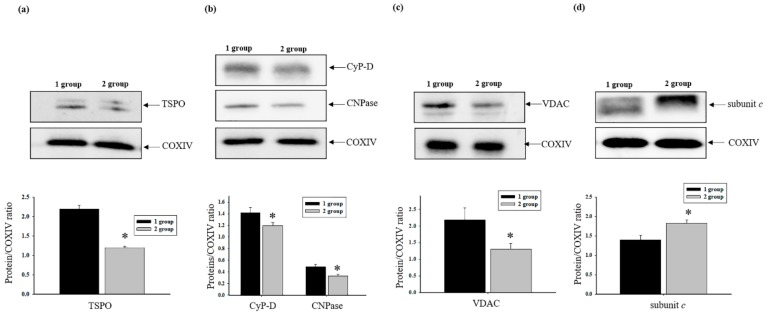
The effect of AST administration on mitochondrial protein content: cyclophilin D (CyP-D), translocator protein (TSPO), 2′, 3′-cyclic nucleotide 3′-phosphodiesterase (CNPase), subunit *c*, and voltage-dependent anion channel (VDAC) in intact RHM from each group of rats. Antibody to cytochrome c oxidase subunit IV (COX IV) was used as loading control. (**a–d**) Upper parts: Western blots stained with corresponding antibodies; (lower parts): diagrams quantitatively reflecting changes in protein content in absolute units normalized to COX IV. Data are presented as means ± SDs of five independent experiments. * *p* < 0.05—significant difference in protein level compared with control (RHM isolated from first group of rats).

Taking into consideration that regulator proteins play an important role in mPTP, in the present study, we examined how AST administration influences the levels of proteins such as CyP-D, TSPO, CNPase, subunit *c*, and VDAC in native RHM isolated from each group. The upper parts of Figure 4a–d show western blots of the proteins in RHM isolated from each group of rats. Quantitative analysis of protein levels is shown in the lower parts of Figure 4a–d. Protein bands were quantified after normalization with respect to cytochrome c oxidase subunit IV (COX IV). As seen from Figure 4a, the TSPO level decreased by 20% in RHM isolated from the second group of rats compared to the first group of rats. After AST treatment, CyP-D and CNPase levels diminished by 15.5% and 32.6%, respectively, in RHM related to control (Figure 4b). In RHM isolated from the second group, the VDAC level decreased 1.6 times (Figure 4c), whereas the level of subunit *c* increased by 30% (Figure 4d) in comparison with the first group of rats.

### 3.5. The Effects of AST Administration on Respiratory Activity, CRC, and Mitochondrial Swelling in Rat Heart Mitochondria

**Figure 5:**
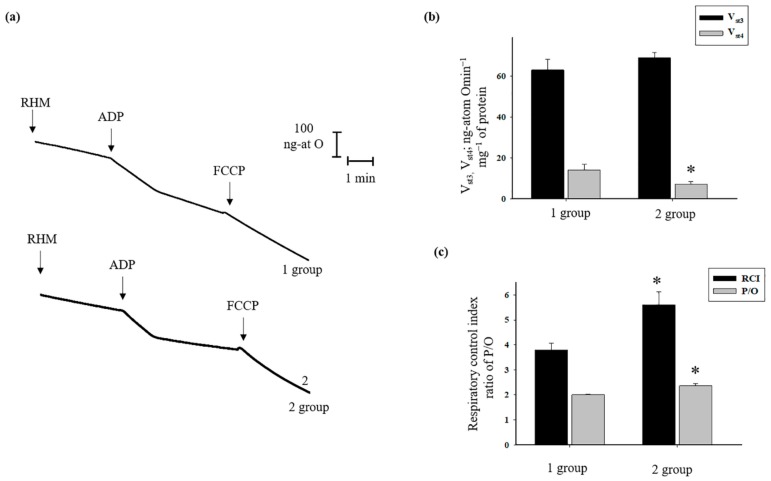
The effects of AST administration on respiratory activity in RHM isolated from each group. RHM were incubated in standard medium, as described in Materials and Methods. (**a**) Curves of respiratory activity; (**b**) quantitative analysis of RHM respiration rate in states 3 and 4; (**c**) RCI and P/O values. Data are presented as means ± SDs of five independent experiments. * *p* < 0.05 compared with control (first group).

To check the inhibitor effect of AST on mPTP functioning, we measured respiratory activity, CRC, and mitochondrial swelling in RHM isolated from two group of rats after AST administration (see Material and Methods). Therefore, the next step in our investigation was to measure the oxygen consumption rates in states 3 and 4, RCI, and P/O in RHM isolated from both groups of rats (Figure 5). Figure 5a shows representative curves of respiratory activity of RHM isolated from each group of rats. According to the experimental data, no substantial change was observed in the oxygen consumption rate in state 3 in RHM from the second group of rats compared to the first group. However, the oxygen consumption rate in state 4 in RHM from the second group of rats slowed down by 50% compared with the first group (Figure 5b). The RCI of RHM from the second group increased by 40% and was approximately 5.36, whereas the RCI of RHM from the first group was 4 (Figure 5c). The P/O ratio in RHM from the first group was 2, whereas in RHM from the second group it was 2.36, increasing by approximately 18%.

**Figure 6:**
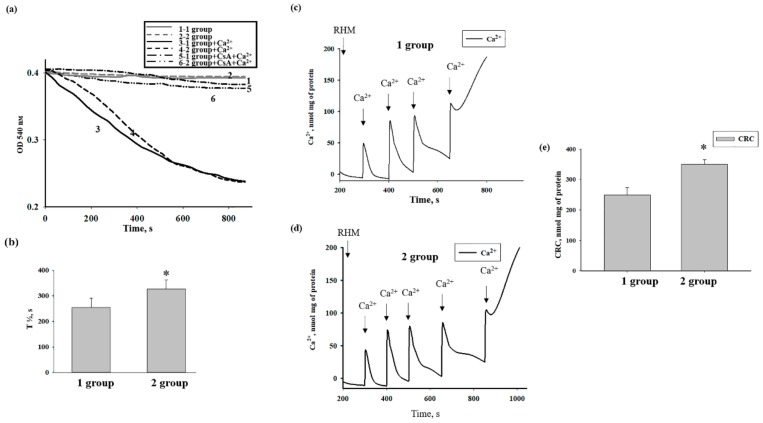
The effect of AST administration on mitochondrial swelling and CRC in RHM isolated from each group. (**a**) Curves of RHM swelling; (**b**) half-maximum of mitochondrial swelling (T_1/2_); (**c**,**d**) Ca^2+^ transport in RHM isolated from each group; (**e**) quantitative analysis of CRC in RHM.

Then, we compared the swelling of RHM isolated from every group of rats. Figure 6a shows representative curves of Ca^2+^-activated swelling of RHM isolated from all groups of rats. Figure 6b demonstrates the average half-maximum (T_1/2_) of mitochondrial Ca^2+^-activated swelling. The half-maximum of the mitochondrial swelling of AST-treated rats increased by 30% (curve 4 versus 3). Thus, the rate of RHM swelling became slower compared to control. CsA inhibited mitochondrial swelling in RHM isolated from each group of rats.

The effect of AST administration on the mPTP function of RHM was examined. Ca^2+^ pulses were added to the mitochondria to reach a threshold Ca^2+^ concentration for mPTP opening as described in Figure 2. In RHM from each group, the first addition of Ca^2+^ led to an active accumulation of Ca^2+^ in mitochondria with subsequent restoration (Figure 6c,d). The release of accumulated Ca^2+^ (mPTP opening) occurred after the fourth and fifth Ca^2+^ additions in control and AST-treated groups, respectively. Figure 6e demonstrates quantitative changes in Ca^2+^ retention capacity in Ca^2+^-loaded RHM isolated from every experimental group of rats. We observed that AST administration increased Ca^2+^ retention capacity by 35% in RHM compared to control.

### 3.6. Changes in the Phosphorylation States of Activated CREB and Akt in Rat Heart Mitochondria after AST Administration during mPTP Opening

**Figure 7:**
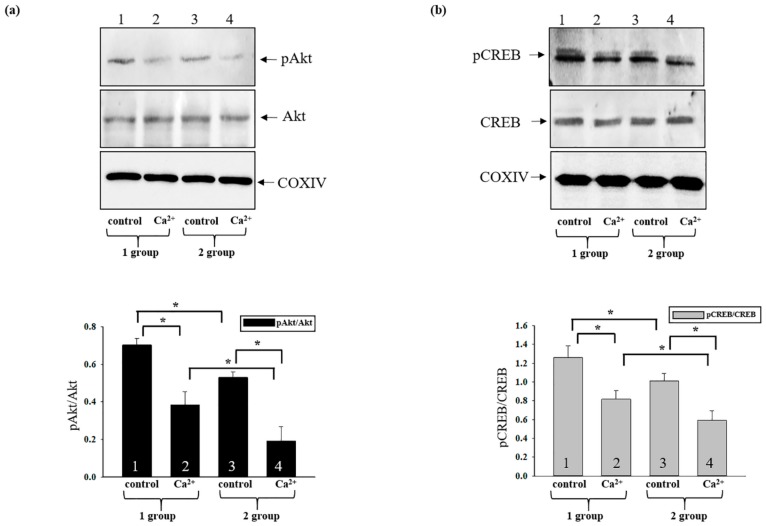
The effects of AST administration on pAkt and pCREB levels during mPTP opening. (**a**,**b**) Upper parts: Western blots stained with corresponding antibodies; lower parts: ratios of pCREB to total CREB, and pAkt to total Akt. COX IV was used as a loading control. Data are presented as means ± SDs of five independent experiments. * *p* < 0.05—significant difference in protein level compared with corresponding control.

It is known that CREB is involved in signaling pathways, and that Ca^2+^ is capable of activating several kinase pathways in order to phosphorylate CREB at Ser133. CaMKI, II, and IV; p70S6K; rsk2; MSK; PKCs; Ras/Raf/MAPK; Akt; and MAPKAP-K2 are capable of phosphorylating CREB, facilitating interaction with the CREB-binding protein (CBP/p300) [44]. In addition, Akt (known as protein kinase B) directly affects mitochondria, protecting them from oxidants or mPTP opening [28]. Therefore, we examined how the levels of phosphorylated Akt and CREB changed in the presence of AST when mPTPs were open (Figure 7). The upper parts of Figure 7a,b show western blots stained with antibodies to pAkt, Akt, pCREB, and CREB. COX IV was used as a loading control. A quantitative analysis of pAkt/Akt and pCREB/CREB ratios is presented in the lower parts of Figure 7a,b. It is visible from the figure that the pAkt/Akt ratio decreased by ~46% upon the induction of mPTP opening, whereas the pCREB/CREB ratio decreased by ~36% compared with control (column 2 versus 1). After AST treatment, the pAkt/Akt and pCREB/CREB ratios decreased by ~25% and ~20% respectively, compared to control (column 3 versus 1). After the addition of the threshold [Ca^2+^] to RHM from AST-treated rats, the pAkt/Akt ratio diminished by 50%, while the pCREB/CREB ratio decreased by ~27% (column 4 versus 2). It should be noted that when mPTP was opened, AST decreased the pAkt/Akt ratio by ~65% and the pCREB/CREB ratio by ~27% compared with the effect of AST alone (without Ca^2+^) (column 4 versus 3).

## 4. Discussion

Mitochondrial dysfunction induced by oxidative damage causes morphological and functional changes in mitochondria. In pathological states, structural changes occurring upon oxidative damage, such as swelling, fragmentation of mitochondria, and division of mitochondria, become more pronounced [41]. Oxidative stress in mitochondria can induce membrane permeabilization and the opening of nonspecific mPTP, which is considered the initial stage of apoptosis [6,45]. mPTP participates in the physiological calcium-release mechanisms that are required for proper metabolic regulation of the cell; this structure could be an important player in the regulation of heart development [46]. AST can diminish oxidative stress and maintain the integrity of mitochondria, as well as sustain mitochondrial function, protecting their redox balance [47]. In addition, it was shown that AST significantly decreases physiologically-arising oxidative stress and maintains mitochondria in a more reduced state even after stimulation with H_2_O_2_; it prevents a drop in membrane potential and increases oxygen consumption by mitochondria [48,49]. Park and coauthors showed that AST treatment increased the mitochondrial content, ATP production, and activity of respiratory chain complexes [50]. In our experiments, we observed that in the presence of both AST added to RHM and AST administration, the RCI and P/O ratio increased (Figure 1 and Figure 5).

Recently, we showed that the addition of melatonin at concentrations of 10 nM and 100 nM to isolated rat-brain mitochondria triggered mPTP opening [51], while chronic administration of melatonin slowed down mPTP opening [52]. Here, we demonstrated that Ca^2+^-induced mPTP opening in isolated RHM was delayed by 5 µM AST. The AST was able to suppress Ca^2+^-induced Ca^2+^ efflux and membrane potential dissipation and increase CRC by 26%. Mitochondrial swelling decreased by 36% (Figure 2 and Figure 3). Moreover, AST administration increased the CRC of RHM by 35%, while the rate of mitochondrial swelling decreased by 30%. AST prevented mitochondrial swelling and detained Ca^2+^ release from RHM both with the direct addition of AST and after AST administration (Figure 6). This result reveals the involvement of AST in mPTP functioning and supports the data in the literature showing an inhibitory effect of AST on oxidative stress-induced mitochondrial dysfunction in living cells [53].

TSPO is localized in the outer mitochondrial membrane at contact sites between the outer and inner membranes [54]. The role of TSPO in the heart has not been completely understood; however, it is known that the protein is implicated in the pathophysiology of cardiac diseases and its ligands improve heart function, which permits consideration of TSPO as a potential target for therapy of cardiovascular diseases [55]. The level of TSPO in the heart varies under different stress conditions; under chronic stress, the level decreases, whereas under acute stress, it increases [55]. Our study showed that the TSPO level in isolated RHM decreased, probably due to the inhibitory effect of AST treatment. (Figure 4a). TSPO can form a multimeric complex with VDAC (which is retained in a purified VDAC preparation [56]). Genetic studies have suggested that the composition of mPTP does not require VDAC [57]. However, VDAC can regulate the rate of Ca^2+^ entry into to the intermembrane space [42], thereby participating in the regulation of mPTP. The decline of VDAC content in RHM isolated from AST-treated rats suggests a decreased rate of Ca^2+^ influx, and therefore slower mPTP opening.

CyP-D is a mitochondrial matrix protein considered a structural component and regulator of mPTP, and is a prominent mediator of mPTP. mPTP, as regulated by CyP-D, appears to be a physiological Ca^2+^ release mechanism that is required for proper metabolic regulation within the mitochondria [58]. The loss of CyP-D does not prevent mPTP opening, but increases the Ca^2+^ load required before opening occurs [59]. A decline of CyP-D content in RHM isolated from AST-treated rats (Figure 4b) could lead to an increased Ca^2+^ load and slower mPTP opening (Figure 6). CyP-D directly binds the lateral stalk of ATP synthase and alters its activity [60]. CyP-D may control the assembly of the electron transport chain, making it a central node for the control of mitochondrial function [61]. Moreover, CyP-D interaction decreases ATP synthesis and hydrolysis rates to modulate both energy production and necrotic cell death [62]. Subunit *c* of the F_o_ sector of F_o_F_1_-ATPase is speculated to be a principal component of the mPTP complex. Subunit *c* plays a critical role in the formation of the Ca^2+^-induced mPTP channel [13,63,64]. In the presence of threshold [Ca^2+^], dephosphorylated subunit *c* has the ability to promote mPTP opening and induce mitochondrial swelling, and lower Ca^2+^ uptake capacity and Δψm. The level of subunit *c* decreased in mitochondria [13]. Here, in RHM isolated from AST-treated rats, the subunit *c* content increased, which contributed to increased Ca^2+^ capacity and a slowing of mitochondrial swelling.

Recently, we showed that CNPase was co-precipitated with CyP-D, ANT, and VDAC as well as a-tubulin in Ca^2+^-loaded and control mitochondria, indicating a possible physical association between these proteins in mitochondria [8]. In particular, CNPase, residing on the outer membrane, could tightly interact with VDAC, determining the permeability of the outer mitochondrial membrane. VDAC channels can be in an open or closed state. In the VDAC closed state, its channels are more permeable for Ca^2+^ [65], which might lead to acceleration of mPTP opening. Both VDAC and CNPase bind to α-tubulin. Binding of α-tubulin to VDAC promotes its closure [66]. Thus, CNPase might regulate VDAC conductance directly or through α-tubulin binding, followed by modulation of outer membrane permeability in mitochondria. Threshold [Ca^2+^] loading promotes rearrangement of mPTP regulators/modulators, which may cause mPTP opening. The decreased level of VDAC in RHM from AST-treated rats may be a reason for changes in the sensitivity to Ca^2+^ upon the AST-evoked inhibition of mPTP in RHM.

Akt is a key participant in the regulation of cellular signals, which are important for cell death and survival [67]. Akt directly affects mitochondria, protecting them from oxidants or mPTP opening [28,68]. VDAC is considered to be a direct substrate of pAkt [69]. Akt is present in mitochondria, and the level of Akt in mitochondria is dynamically regulated by cellular signaling activities. In mitochondria, Akt phosphorylates the β subunit of ATP synthase, GSK-3β, and the mitochondrial form of hexokinase II, which promotes the association of hexokinase II with VDAC [70]. The result of this association is decreased conductivity of VDAC. Akt has been shown to phosphorylate VDAC in isolated heart mitochondria [71]. The results of our observations show that inactivation of pAkt could lead to a reduction in the VDAC level and inhibition of mPTP.

Wang and coauthors showed that AST was able to activate the cAMP/PKA/CREB signaling pathway in brain tissues [72]. Recently, we showed that CNPase was phosphorylated by PKA in rat brain mitochondria [73]; thus, CNPase could be a target of the AST effect in RHM. Moreover, CREB is a downstream substrate of Akt. Phosphorylation of CREB by Akt at Ser133 leads to activation of its transcription and inhibition of apoptosis [32]. Activated CREB inhibits mitochondrial activity, impairing the mitochondrial state [37]. Our results show that AST decreased CREB signaling and improved the mitochondrial state. Because mPTP is considered to be the initial stage of apoptosis [45] and the addition of AST inhibited the induction of mPTP opening, activation of Akt and CREB being inhibited in this case, it may be suggested that AST is capable of improving RHM functions, which constitute an important factor in the normal functioning of the heart. These data provide the impetus for further studies of the therapeutic potential of AST in the treatment and prevention of heart diseases.

## 5. Conclusions

To summarize, the results of the study suggest that AST is capable of improving the functional state of RHM, increasing RCI and P/O ratios both with direct addition of AST to RHM and AST administration. AST, a dietary carotenoid, is considered a mitochondrion-permeable antioxidant [49] that can penetrate the mitochondria, and is effective in preventing mPTP opening. TSPO and its ligands activate oxidative stress and apoptosis [74], which can lead to mPTP opening. TSPO interacts with VDAC and is capable of forming a firm complex with it [56]. CNPase, residing on the outer membrane, can tightly interact with VDAC, determining the permeability of the outer mitochondrial membrane [8]. AST treatment reduced the levels of TSPO, VDAC, and CNPase in native RHM, thereby detaining mPTP opening. A decreased content of CyP-D in RHM isolated from AST treated rats can lead to an increased content of subunit *c* and Ca^2+^ capacity, thereby slowing down mPTP opening. AST treatment could delay Ca^2+^-induced Ca^2+^ release. AST administration blocked activated Akt in RHM. Phosphorylation of CREB (as a downstream Akt substrate) by Akt resulted in its transcriptional activation and inhibition of apoptosis [32,75]; we observed that the level of activated CREB was attenuated by AST administration. Since AST improves the resistance of RHM to Ca^2+^-dependent stress, it can be assumed that after further studies, this antioxidant can be considered an effective tool for improving the functioning of the heart muscle in general under normal and clinical conditions. Determining the effect of AST on cardiomyocyte cells and investigating the functional state of RHM after chronic administration of AST in heart failure are the aims of our further studies.
